# Patients’ perceptions toward and the driving factors of decision-making for opportunistic bilateral salpingectomy at the time of cesarean section

**DOI:** 10.4274/tjod.galenos.2020.12129

**Published:** 2020-07-29

**Authors:** Murat Yassa, Çiğdem Pulatoğlu

**Affiliations:** 1 Sancaktepe Şehit Prof. Dr. İlhan Varank Training and Research Hospital, Clinic of Obstetrics and Gynecology, İstanbul, Turkey; 2 İstinye University, Medical Park Gaziosmanpaşa Hospital, Clinic of Obstetrics and Gynecology, İstanbul, Turkey

**Keywords:** Opportunistic salpingectomy, permanent contraception, postpartum sterilization, prophylactic salpingectomy, risk-reducing salpingectomy

## Abstract

**Objective:**

Enough data can be found in the literature regarding the protective effect of tubal ligation on gynecological cancers. In addition, a large body of evidence revealed that prophylactic bilateral salpingectomy had no significant negative effect on the ovarian function, quality of life, sexuality, surgery duration, and cost-effectivity. This study was aimed at exploring the underlying factors that motivate women for either opportunistic bilateral salpingectomy (OBS) or tubal ligation, particularly focusing on their preferences, knowledge, and beliefs toward female sterilization, satisfaction from counseling, and body image following the salpingectomy.

**Materials and Methods:**

A total of 54 patients who had undergone surgical sterilization with either OBS or tubal ligation were included in this prospective cohort study. The acceptance rate of the OBS at the time of cesarean section among pregnant women seeking surgical sterilization was calculated. The underlying reasons for women’s acceptance or refusal for salpingectomy were assessed by a non-validated data collection tool that had 14 open-ended questions focusing on the women’s preferences, knowledge, beliefs toward female sterilization, satisfaction from counseling, and body image following the salpingectomy.

**Results:**

The acceptance rate of OBS at the time of cesarean section among pregnant women and electively among non-pregnant women were 93.5% (n=43/46) and 75% (6/8), respectively. The main driving factors influencing the decision of preferring OBS over tubal ligation were the risk-reducing effect for ovarian cancer and superior pregnancy prevention.

**Conclusion:**

The acceptance rate of OBS at the time of cesarean section was found to be very high, and it should therefore be offered at the time of cesarean section to women who desire permanent contraception.

**PRECIS:** Obstetricians should be eager to offer opportunistic bilateral salpingectomy (OBS) at the time of cesarean section to women desiring permanent contraception following a detailed counseling of its potential benefits. The most common motivators of women for consenting to an OBS procedure were risk-reducing potential for ovarian cancer and superior pregnancy prevention.

## Introduction

Tubal sterilization either with tubal occlusion or ligation and mid-isthmic partial salpingectomy is one of the most popular and effective methods of permanent contraception worldwide^(1)^. More than one-fifth women in the United States undergo a surgical female sterilization as a method of contraception^(2)^. Postpartum tubal ligation is performed after approximately 8-9% of all births. The cumulative 10-year probability of pregnancy is as low as 0.75%^(3)^.

There are now solid data about the protective effect of tubal ligation on gynecological cancers. A pooled systematic review and meta-analysis of all types of tubal procedures showed that tubal sterilization reduced the endometrial cancer risk approximately by 42%^(4)^. Contrary to the endometrial cancer, epithelial ovarian cancer lacks an effective screening method and is the leading cause of mortality due to gynecological cancers in the developed countries, and the second highest globally^(5)^. It has been hypothesized previously that the fallopian tubes are very likely to be the origin of high-grade serous cancers^(6)^, and thus, prophylactic or opportunistic salpingectomy at the time for hysterectomy and other benign procedures may be beneficial. The incidence of ovarian cancer among women who had undergone prophylactic salpingectomy along with hysterectomy for benign condition was found to be reduced to 30-64%^(7)^. In addition, a large body of evidence revealed that prophylactic bilateral salpingectomy had no significant negative effect on the ovarian function, quality of life, sexuality, surgery duration, and cost-effectivity^(7)^.

Prophylactic salpingectomy is a cost-effective and feasible strategy recommended for reducing the risk of ovarian cancer at the time of gynecologic surgery in women past childbearing age^(8)^. A similar body of evidence for opportunistic approach at cesarean section is also growing. It has been calculated that opportunistic salpingectomy would lead to approximately 17 fewer ovarian cancer diagnoses, 13 fewer ovarian cancer deaths, and 25 fewer unwanted conceptions compared to tubal ligation for every 10,000 opportunistic salpingectomy at the time of cesarean section^(9)^.

However, salpingectomy refers to the surgical removal of a female reproductive organ. Some women may have apprehensions with respect to salpingectomy due to religious concerns, reduced self-image, or tubal re-anastomosis, and the decision-making process might be influenced by sociodemographic features and lack of knowledge^(10,11,12)^.

The investigators have experienced denials from women who were seeking tubal ligation as a sterilization procedure during cesarean section after a comprehensive counseling for prophylactic salpingectomy. It was aimed to explore the underlying factors that motivate women for either opport unistic bilateral solpingectomy (OBS) or tubal ligation, particularly focusing on their preferences, knowledge and beliefs toward female sterilization, satisfaction from counseling, and body image following the salpingectomy.

## Materials and Methods

This descriptive cohort study was conducted at a secondary center between February and June 2019 and included women who agreed to surgical sterilization with either OBS or tubal ligation. The study was approved by the local administration board and registered with the National Clinical Trials Registry (NCT #03830502). The study were approved by the University of Health Sciences Turkey, Şişli Hamidiye Etfal Training and Research Hospital Local Ethics Committee (approval number: 1182, date: 05/03/2019). Data were prospectively collected and retrospectively analyzed. The study included pregnant and non-pregnant women aged >18 years who either electively or at the time of cesarean section gave their consent for surgical sterilization. However, women with category-1 CS, clinical conditions that lead to planned cesarean hysterectomy such as placenta percreta, a history of ovarian cancer, previous chemotherapy or radiation, those who had previously undergone sterilization or withdrawn their consent prior to the surgery or whose surgical procedure could not be completed were excluded from the study. Once the patients’ desire for sterilization was confirmed, surgical sterilization was discussed with the patients in the presence of indications. After the 32^th^ gestational week, pregnant women were initially approached with OBS during cesarean section as a primary surgical sterilization procedure. While a standard bilateral salpingectomy was performed in those who gave consent for salpingectomy, a tubal ligation with Pomeroy technique was performed in those who did not. For nonpregnant women, a laparoscopic tubal ligation was proposed rather than a hysteroscopic procedure due to technical and financial reasons. Further, pregnant women who had vaginal birth were proposed immediate contraception with intrauterine device or oral contraceptives; however, they were irrelevant to the objective of this study. While the primary outcome of the study was the acceptance rate of OBS at the time of cesarean section among pregnant women who seek surgical sterilization, the secondary outcomes were the patients’ multifaceted perceptions toward sterilization and OBS and the driving factors behind the decision-making for OBS at the time of cesarean section. The secondary outcomes were measured using a non-validated data collection tool with 14 open-ended questions assessing the factors behind the decision of salpingectomy or tubal ligation (Table 1). The data collection tool did not have a scoring or a range and questioned the underlying reasons for women’s acceptance or refusal to salpingectomy in detail, focusing on their preferences (Q1, 4, 9-12), knowledge (Q2, 3, 7, 8) and beliefs (Q5, 6) toward female sterilization, satisfaction (Q13) from counseling, and body image following the salpingectomy (Q14). Finally, the open-ended answers were combined under similar answers. The income of the women were scaled between 1 and 3 (1: low-, 2: middle-, 3: high-income), and their occupations were scaled between 1 and 4 (1: unemployed, 2: worker, 3: government employee, 4: tradesmen/craftsmen). Informed consent was obtained.

### Statistical Analysis

The data collected through the questionnaires were analyzed using the IBM SPSS Statistics (version 22.0; IBM Corporation, Armonk, NY). The demographic variables and specific scale measures were then presented as mean, standard deviation, standard error of mean, median, interquartile range (IQR), and frequency for the relevant items.

## Results

**Figure 1:**
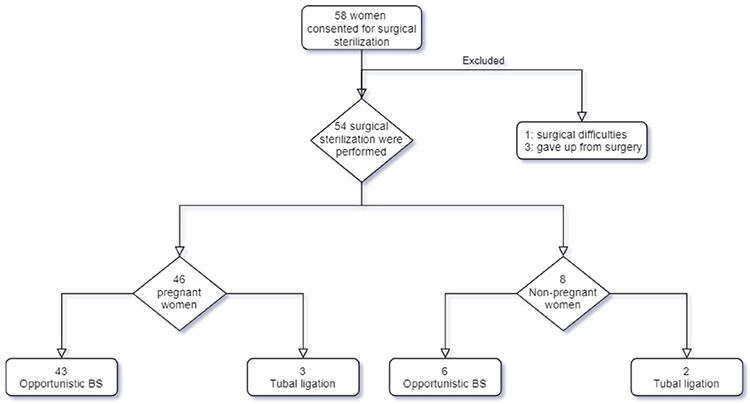
Flowchart of the women included in the study

A total of 58 women agreed to undergo a sterilization surgery and gave their consent for it. However, four women were later excluded from the study as three of them withdrew their consent for sterilization prior to the procedure and one of them had extensive adhesions due to which the procedure was abandoned in order not to increase the morbidity in the patient. Finally, a total of 54 surgical sterilization were performed (Figure 1).

The mean age of the women was 37.9±1.8 years and ranged between 34 and 42 years. The mean body mass index was 28.9±3.8 and ranged between 21 and 40. While the median parity was 3 (IQR: 2, minimum: 1, maximum: 6), the median income of the families was medium-income and the median occupation of the partner was governmental employee.

The acceptance rate of OBS at the time of cesarean section among pregnant women and electively among non-pregnant women were 93.5% (n=43/46) and 75% (6/8), respectively.

The answers to the questions regarding the preferences (Q1, 4, 9-12), knowledge (Q2, 3, 7, 8) and beliefs (Q5, 6) toward female sterilization, satisfaction (Q13, 14) from counseling, and body image following the salpingectomy (Q15) were schematized (Figures 2, 3, 4, 5, 6, 7, 8, 9, 10, 11, 12, 13, 14, 15).

## Discussion

This study revealed that 91% of the participating women overwhelmingly preferred OBS over a standard tubal ligation. The women who preferred tubal ligation over OBS did so due a lack of knowledge about the procedure and further menstruation irregularities, possibility of re-opening of the tubes in the future, reduced body image, and influence of the partner. The main driving factor behind the decision preferring OBS over tubal ligation was the risk-reducing effect for ovarian cancer in 63% of the patients. The second most common motivation was superior pregnancy prevention in 19% of the women.

A recent survey study of all women seeking permanent contraception revealed a high accepting OBS rate of 63% in pregnant and 85% in nonpregnant women^(11)^. Comparable to our results, the two main motivational factors for choosing salpingectomy were superior pregnancy prevention and risk reduction in ovarian cancer with 61% and 33%, respectively^(11)^.

OBS at the time of hysterectomy or interval sterilization has become a routine practice for reducing the risk of ovarian cancer. In 2015, an American College of Obstetricians and Gynecologists Committee Opinion recommended that obstetricians should discuss the possible risk-reducing benefits of bilateral salpingectomy with patients who wish to have permanent contraception^(13)^. However, the embracement of this strategy at the time of cesarean delivery for pregnant women who desire permanent sterilization has not been widely adopted, probably due to a lack of available data including the surgical and psychological data in this setting^(14)^. By 2017, the overall proportion of salpingectomies in patients who underwent a permanent contraception procedure was reported to be as high as 61.5%, in a nationwide data analysis^(15)^. In a large retrospective cohort study of seven years, almost half of the women preferred OBS as the mode of contraception at either elective or unscheduled cesarean section, which implies that OBS has a high acceptable rate for its higher contraceptive efficacy and risk-reduction benefit for ovarian cancer^(16)^.

About one-fifth of the women reported that they did not have enough information about other contraception methods prior to our detailed counseling, half of the women were not informed properly about the future recanalization of ligated fallopian tubes, 96% of the women were familiar with the options that were available following a salpingectomy should they desire fertility in future, and about 57% of them had accurate information about the success rates of future assisted reproductive techniques. These results enlightened the authors about the importance of the detailed counseling prior to suggesting the OBS option and, more importantly, encouraging the clinicians about non-inferiority of the procedure to comply with this risk-reducing strategy. A survey among physicians performing OBS reported that 46% of the surgeons had barriers such as suspicions about increased complications, decreased ovarian reserve, and increased counseling time while performing salpingectomy along with hysterectomy^(17)^. Therefore, a fully detailed counseling on the major advantages of OBS is crucial, following which, 96% of the women in our study reported being satisfied.

Of the women who preferred a tubal surgery in the current study, about 90% either had a contraindication for the use of other oral or intrauterine methods, or experienced side effects, or had difficulties on their regular use. Almost 60% of them sought permanent contraception due to financial difficulties and 30% expressed their concerns on raising more than one child. Although the women with two different answers surely had common thoughts, the latter may have underlying social and lifestyle difficulties that has to be investigated in future studies.

Interestingly, 70% of the women participating in this study were not sure if tubal ligation and removal of tubes are a sin or not. The authors could not find any related information in the literature while writing this article. However, despite that knowledge gap, majority of the women preferred salpingectomy. Although the authors have postulated that it might be related to their idea of body image, 95% of the women stated that they did not feel incomplete following the removal of fallopian tubes and losing their fertility. Determining the reasons and developing a strategy to overcome this issue and to increase the OBS rates are certainly the matters of future research.

A recent systematic review and meta-analysis of OBS at the time of cesarean section in women who underwent permanent sterilization revealed that although the bilateral salpingectomy slightly increased the operative time, it was comparable to tubal ligation in terms of complications, completion rate, and short-term ovarian reserve with a greater cost-effectiveness^(18)^. Another larger and more recent systematic review and meta-analysis determined similar results in which OBS was not associated with more adverse outcomes than tubal ligation^(19)^. Providing those favorable data to the women who seek permanent contraception might have affected the high consent rates for OBS in the current study.

However, while tubal sterilization is a highly effective method of contraception, its effectiveness varies by the surgical method, and the prevalence of regret has been reported to be between 0.9 and 26% with a cumulative probability of 12.7%^(20)^. Therefore, the future regret rates should be carefully assessed to better inform patients about the local circumstances. Authors postulated that the mean age of 38 years in the current study will probably reduce the regret rates, although they currently do not have the data.

Prophylactic and OBS is an increasing trend among obstetricians and has also proven to be an effective risk-reducing method for ovarian cancer. Future studies should focus on the underlying reasons behind last-minute refusals, the rates and features of unmet contraception needs, and the rates and outcome of patients who desire fertility again in the future following the OBS at the time of cesarean section.

### Study Limitations

One of the limitations of the study is the small number of cases. The other limitation is the lack of data related to other factors like education, underlying reasons of last-minute refusals, the rates and features of unmet contraception needs which may also affect the decision of patients.

## Conclusion

The acceptance rate of OBS at the time of cesarean section was found to be very high. The main driving factor behind the decision of preferring OBS over tubal ligation was its risk-reducing effect for ovarian cancer and superior pregnancy prevention. Obstetricians are recommended to take every chance of offering OBS at the time of cesarean section to women who desire permanent contraception.

## Figures and Tables

**Table 1:**
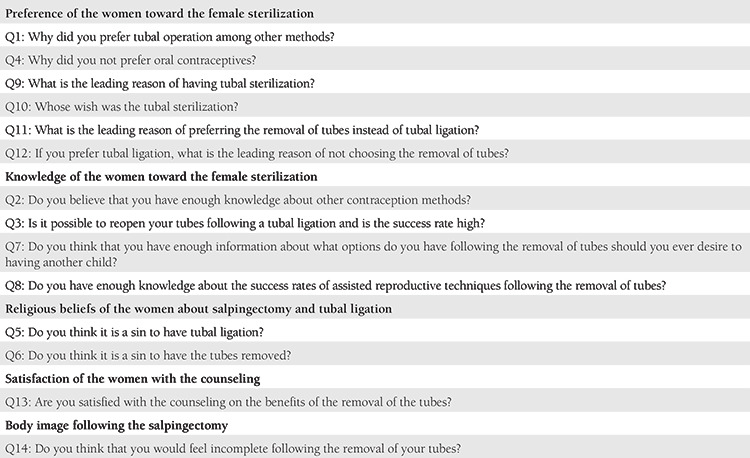
Outline of the questionnaire

**Figure 2:**
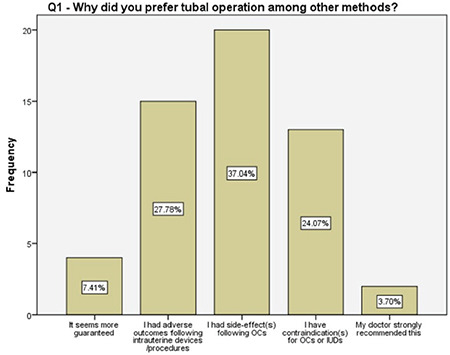
The reason for women’s preference of tubal surgery

**Figure 3:**
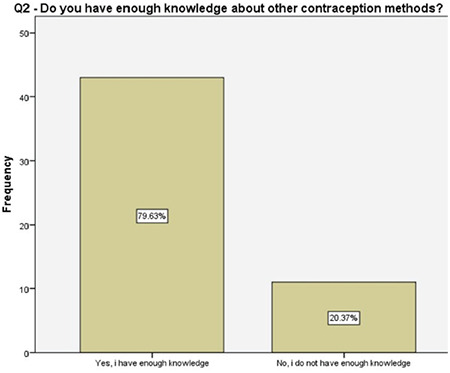
Women’s knowledge of other contraception methods

**Figure 4:**
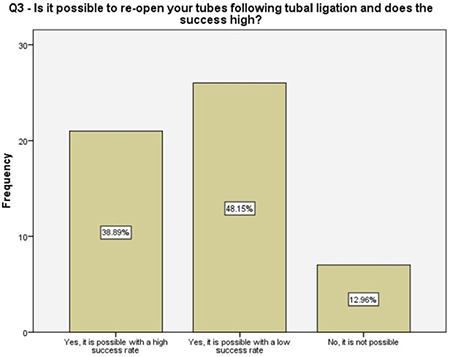
Women’s knowledge toward the reversal of tubal ligation

**Figure 5:**
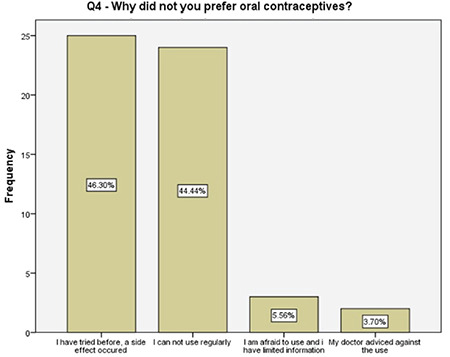
Underlying reasons for not choosing oral contraceptives

**Figure 6:**
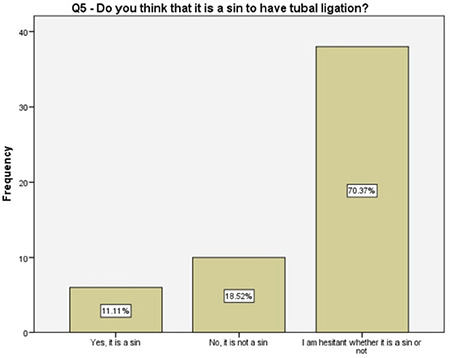
Women’s religious beliefs toward the salpingectomy

**Figure 7:**
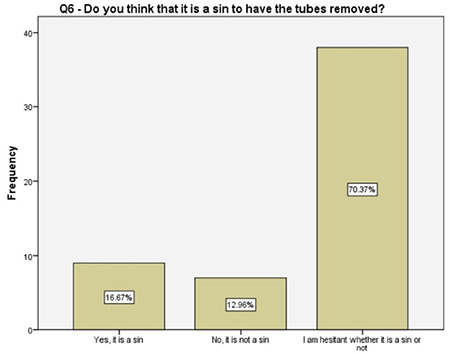
Women’s religious beliefs toward the tubal ligation

**Figure 8:**
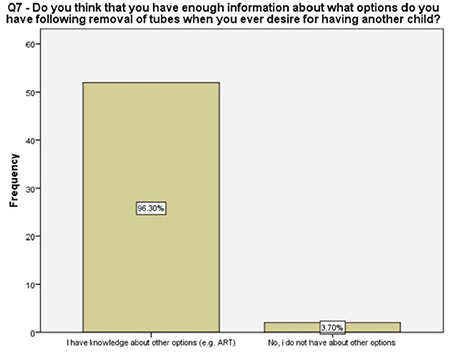
Women’s knowledge of the future treatment options following the salpingectomy

**Figure 9:**
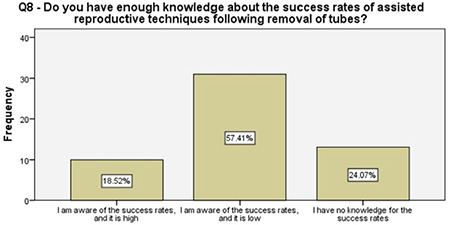
Women’s knowledge of the success for future ART following the salpingectomy

**Figure 10:**
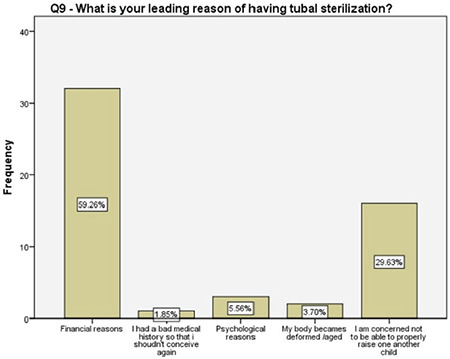
Women’s main motivation for having tubal sterilization

**Figure 11:**
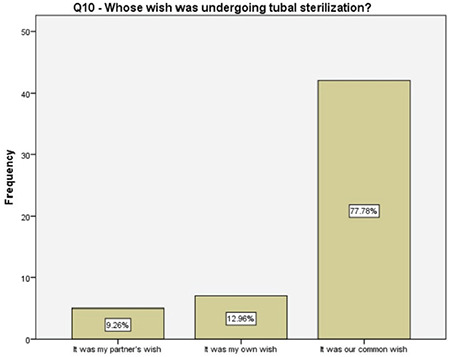
Decision-making on having tubal sterilization

**Figure 12:**
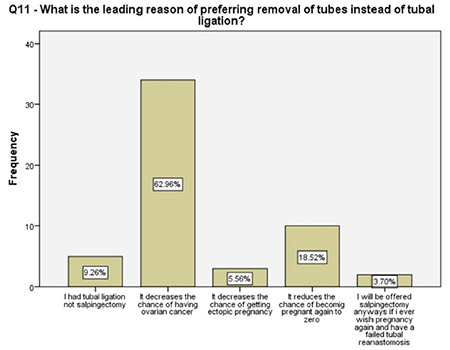
Women’s main motivation for having salpingectomy instead of tubal ligation

**Figure 13:**
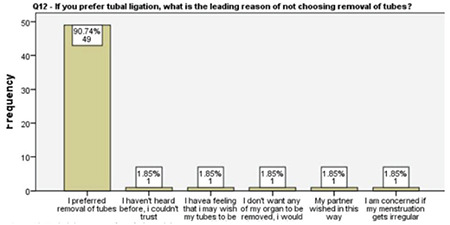
Underlying reasons for refusing salpingectomy

**Figure 14:**
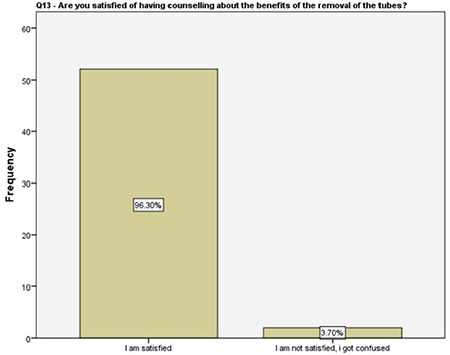
Women’s satisfaction with the detailed counseling on salpingectomy

**Figure 15:**
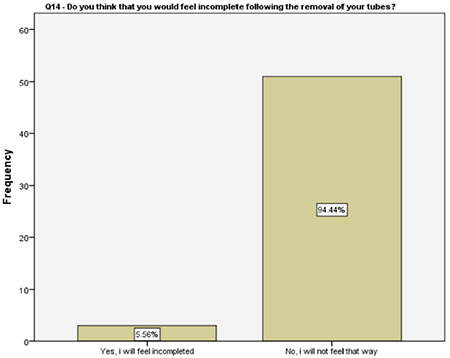
Women’s body image upon salpingectomy
